# Myrtol

**Published:** 1889-04

**Authors:** 


MYRTOL, that part of the oil of myrtha which boils at 160, given in gelatine capsules containing one minim, two or three capsules daily, removes all fetor of the breath in putrid bronchitis, and seems to possess even curative effect, while it does not influence phthisis.

